# Supplementary material for: Epidemiological trends of Lassa fever in Nigeria, 2018–2021
Source: PLoS One. 2022 Dec 30;17(12):e0279467. doi: 10.1371/journal.pone.0279467 (PMC9803109; doi:10.1371/journal.pone.0279467)
Supplement: Attachment — Submitted filename: Lassa Fever Review Report.docx [file pone.0279467.s001.docx]

**Topic: Epidemiological Trends of Lassa Fever in Nigeria, 2018 -2021**

**Summary:** The author has described the trends of Lassa fever in Nigeria in the recent years to enhance the public understanding of its epidemiology and possible intervention strategies needed to mitigate the disease burden. The authors analyzed the national surveillance data on the disease obtained from the Nigeria Centre for Disease Control (NCDC), by describing the disease prevalence, sociodemographic, geographic distribution, and the clinical implications of the disease. As a result of this study, the authors recommend as a priority, the sustainability of improved surveillance, enhancement of laboratory diagnosis, and improvement of case management capacity during off-peak periods. Lassa fever is one of the deadly endemic diseases that are of human health burden in some African countries, as a result, studies like this are needed to provide more knowledge on the spread and control of the infection in the human population. With this being said, I recommend this work for publication in this reputable journal after some minor corrections.

**Background - Page 2**

1. Line 25: The first statement should be reconstructed. Are the authors intending to say that **Lassa fever is a viral hemorrhagic fever which is endemic in Nigeria**?
2. Line 25-Line 26: The second statement from Line25 should be rephrased. The authors need to be conscious of the sentences meaning. Here, the intention is to inform readers that improved surveillance and testing capacity has revealed an increase in number of Lassa fever cases. But the sentence here is indicating that it has resulted in an increased number of cases, which is not true.

**Introduction – Page 3-4**

The author should revise the complete manuscript for typos, grammatical errors, and out-of-context sentences. For examples

1. Line 45: The first sentence is incomplete. Emphasize should be made on Lassa fever as an endemic disease, so include “tropical disease which is endemic in West Africa”
2. Line 47: Include the full meaning of RNA before the abbreviation.
3. Line 47 – Line 49: This is a long sentence that will need some punctuation marks.
4. Line 60: Include the abbreviation in a bracket like (ILFR&C), followed by a comma
5. Line 65 – Line 67: This is a long sentence that will need some punctuation marks.
6. Line 81 – 83: The authors should use this section to emphasize on the novelty of this study. There have been literatures on Lassa fever trends, how is this study different and what is the advantage of this study to present knowledge on the disease burden in Nigeria.

**Page 5**

1. Line 98: I will assume the Study design was meant to be a subsection like other are. I will suggest that the Study design and data source should be merged since the writeup in the study design is so limited.
2. Include the full meaning of IDSR before the abbreviation, also in Page 6 Line 126, the LF should be defined before abbreviation.

**Results – Page 8**

1. Line 180 – 181: The authors reported that CFR had significant improvement over the years in high-burden states except for 2021. WHY? Authors should discuss possible cause of this result. This can be discussed from the standpoint of the reported cases, is there any difference in the trends? The epidemiological implications should also be discussed.

**Discussion**

1. Line 256: COVID-19 not COVID 19.
2. The authors have recommended some further studies requirements and implementations that can further reduce the burden of this disease in the human population. However, it is also important to investigate the impact of control interventions and the financial resources available for a response particularly during outbreaks. As a result, the authors should include and refer readers to the following studies on Lassa fever modeling in Nigeria.

- Mathematical analysis of a Lassa fever model in Nigeria: optimal control and cost-efficacy. *Int. J. Dynam. Control* (2022).
- Optimal control and cost-effectiveness analysis for the dynamic modeling of Lassa fever, J. Math. Comput. Sci., 12 (2022), Article ID 136
- Modeling, analyzing, and simulating the dynamics of Lassa fever in Nigeria. *J Egypt Math Soc* **30,** 1 (2022).
- Modeling the dynamics of Lassa fever in Nigeria *J Egypt Math Soc* **29**, 16 (2021).

**General comments**

The author needs to read and revise the complete manuscript as this cannot be accepted in this version. I will recommend this for publication after making the above corrections and suggestions.
