# Supplementary material for: Epidemiological trends of Lassa fever in Nigeria, 2018–2021
Source: PLoS One. 2022 Dec 30;17(12):e0279467. doi: 10.1371/journal.pone.0279467 (PMC9803109; doi:10.1371/journal.pone.0279467)
Supplement: Attachment — Submitted filename: Reviewers Comments.docx [file pone.0279467.s002.docx]

**General comments**:
The paper brought out further evidence of the recent changes to the epidemiology of Lassa in the country, particularly its increased all year round incidence, and a new perspective on the differences in risk groups. Furthermore, being a review of country level secondary data, the paper stimulates further research interests based on some interesting hypothesis.

I believe the paper can be improved by having a paragraph in the background on some prevailing practices in Nigeria which have been linked to increased risk of spread of the disease epidemiology (open drying of grains, backyard farming, poor IPC practices, etc.).

A general review of the background paragraphs would be helpful, particularly in linking it to the study justification: it is understandable, but the flow could be improved in places.

The data source section did not highlight how representative that data is, and if there are some assumptions related to the data sources and what mitigation measures were taken to minimize the effect on the conclusions driven from the results.

The discussion section could be improved by highlighting possible explanation of some of the results (variations in geographic distribution, CFR, etc.). This will further strengthen some of the recommendations proffered.

**Specific issues:**

Lines 49-52. The references seem outdated, with the stated epidemiology not in concurrence with what some recent data reveal.

Line 91-92. You have stated the role of the state and the federal government in health care and surveillance services but did not outline. That of the local government, which has the larger role in coordinating collation of surveillance data from all health facilities.

Line 171-172. Review the sentence. There seems to be a grammatical issue there.

Line 284. The paragraph is bulleted, which is likely a typographic error. Please review.

Line 302-304. The sentence is vague and could be improved by paraphrasing some sections.
