# Supplementary material for: Epidemiological trends of Lassa fever in Nigeria, 2018–2021
Source: PLoS One. 2022 Dec 30;17(12):e0279467. doi: 10.1371/journal.pone.0279467 (PMC9803109; doi:10.1371/journal.pone.0279467)
Supplement: Attachment — Submitted filename: Response to Reviewers.docx [file pone.0279467.s003.docx]

Dear Academic Editor and Reviewers

Thanks for your efforts towards improving the standard our manuscript. We found your comments and suggestions very valuable and look forward to mare support from you.

Below are the comments you made (highlighted in red) and our response immediately below each of the comments. We look forward to your further review and a favourable outcome toward publishing our manuscript in your prestigious journal.

**Academic Editor:**

If applicable, we recommend that you deposit your laboratory protocols in [protocols.io](http://protocols.io/) to enhance the reproducibility of your results. Protocols.io assigns your protocol its own identifier (DOI) so that it can be cited independently in the future

Response: This may not be applicable given that this is a secondary data analysis. However, we have submitted this request to our laboratory team for their consideration. We could upload our Lassa fever data collection tool or laboratory protocol if essential to the peer review process.

1. Please ensure that your manuscript meets PLOS ONE's style requirements, including those for file naming. The PLOS ONE style templates can be found at 
<https://journals.plos.org/plosone/s/file?id=wjVg/PLOSOne_formatting_sample_main_body.pdf> and 
<https://journals.plos.org/plosone/s/file?id=ba62/PLOSOne_formatting_sample_title_authors_affiliations.pdf>

Response: We have cross checked our manuscript to ensure it is in keeping with PLOS ONE’s style requirements as advised

2. In the ethics statement in the manuscript and in the online submission form, please provide additional information about the patient records/samples used in your retrospective study. Specifically, please ensure that you have discussed whether all data/samples were fully anonymized before you accessed them and/or whether the IRB or ethics committee waived the requirement for informed consent. If patients provided informed written consent to have data/samples from their medical records used in research, please include this information.

Response: The ethics statement has been reviewed to include the waiver for informed consent provided by the NHREC to analyse patient data collected during the outbreak by the NCDC (see pages 268 – 272 of the ‘Revised Manuscript with Track Changes). This has been reflected in the online submission form as well.

3. Please ensure that you have specified (1) whether consent was informed and (2) what type you obtained (for instance, written or verbal, and if verbal, how it was documented and witnessed). If your study included minors, state whether you obtained consent from parents or guardians. If the need for consent was waived by the ethics committee, please include this information.

Response: The need for consent was waived by the NHREC as stated in response to (3) above.

4. We note that the grant information you provided in the ‘Funding Information’ and ‘Financial Disclosure’ sections do not match.

When you resubmit, please ensure that you provide the correct grant numbers for the awards you received for your study in the ‘Funding Information’ section.

Response: We have provided the correct grant numbers and cross-checked to ensure there is no mismatch between the information provided in the ‘Funding Information’ and ‘Financial Disclosure’.

5. Thank you for stating the following in the Acknowledgments Section of your manuscript: 
"This research is funded by the Department of Health and Social Care using UK Aid funding and is managed by the National Institute for Health and Care Research. The views expressed in this publication are those of the author(s) and not necessarily those of the Department of Health and Social Care."

We note that you have provided funding information that is not currently declared in your Funding Statement. However, funding information should not appear in the Acknowledgments section or other areas of your manuscript. We will only publish funding information present in the Funding Statement section of the online submission form. 
Please remove any funding-related text from the manuscript and let us know how you would like to update your Funding Statement. Currently, your Funding Statement reads as follows: 
"This research is funded by the Department of Health and Social Care using UK Aid funding and is managed by the National Institute for Health and Care Research. The funders had no influence on the study design, data collection, analysis, decision to publish, or preparation of the manuscript."

Please include your amended statements within your cover letter; we will change the online submission form on your behalf.

Response: We have removed the funding statement from the Acknowledgement section and amended the statements within the cover letter. We appreciate the help of the Editorial Team for changing this aspect in the online submission form on our behalf.

6. Thank you for stating the following in your Competing Interests section:  
"The authors declared no competing interests."

Please complete your Competing Interests on the online submission form to state any Competing Interests. If you have no competing interests, please state "The authors have declared that no competing interests exist.", as detailed online in our guide for authors at <http://journals.plos.org/plosone/s/submit-now> 
 This information should be included in your cover letter; we will change the online submission form on your behalf.

Response: We have reviewed our submission in the competing interest section and have included the amended the statements within the cover letter. We appreciate the help of the Editorial Team for changing this aspect in the online submission form on our behalf.

7. In your Data Availability statement, you have not specified where the minimal data set underlying the results described in your manuscript can be found. PLOS defines a study's minimal data set as the underlying data used to reach the conclusions drawn in the manuscript and any additional data required to replicate the reported study findings in their entirety. All PLOS journals require that the minimal data set be made fully available. For more information about our data policy, please see <http://journals.plos.org/plosone/s/data-availability>.

"Upon re-submitting your revised manuscript, please upload your study’s minimal underlying data set as either Supporting Information files or to a stable, public repository and include the relevant URLs, DOIs, or accession numbers within your revised cover letter. For a list of acceptable repositories, please see <http://journals.plos.org/plosone/s/data-availability#loc-recommended-repositories>. Any potentially identifying patient information must be fully anonymized.

Important: If there are ethical or legal restrictions to sharing your data publicly, please explain these restrictions in detail. Please see our guidelines for more information on what we consider unacceptable restrictions to publicly sharing data: <http://journals.plos.org/plosone/s/data-availability#loc-unacceptable-data-access-restrictions>. Note that it is not acceptable for the authors to be the sole named individuals responsible for ensuring data access.

We will update your Data Availability statement to reflect the information you provide in your cover letter.

Response: We have reviewed our data availability statement and have uploaded the deidentified, cleaned, coded dataset to Zenodo.org repository. Access to the dataset will be obtained through the URL: <https://zenodo.org/record/7309567#.Y23a5XbMI2x>

or

DOI: <https://doi.org/10.5281/zenodo.7309567>

8. Please review your reference list to ensure that it is complete and correct. If you have cited papers that have been retracted, please include the rationale for doing so in the manuscript text or remove these references and replace them with relevant current references. Any changes to the reference list should be mentioned in the rebuttal letter that accompanies your revised manuscript. If you need to cite a retracted article, indicate the article’s retracted status in the References list and also include a citation and full reference for the retraction notice.

Response: We have reviewed the reference list for completeness and stated this in the rebuttal letter.

Review Comments to the Author

Please use the space provided to explain your answers to the questions above. You may also include additional comments for the author, including concerns about dual publication, research ethics, or publication ethics. (Please upload your review as an attachment if it exceeds 20,000 characters)

Reviewer #1: After carefully reading the manuscript, I can say that the manuscript is technically sound, and the data support the conclusions. The data was not made available, however the authors mentioned this can be accessed on the NCDC website.

Response: Data availability has been further clarified

Reviewer #2: The paper brought out further evidence of the recent changes to the epidemiology of Lassa in the country, particularly its increased all year round incidence, and a new perspective on the differences in risk groups. Furthermore, being a review of country level secondary data, the paper stimulates further research interests based on some interesting hypothesis.

Reviewer #3: In general, the paper provides useful information pertaining to the current case distributions for Nigeria with regard to age, geographic distribution, and profession.

General comments:

Lines 266-267: Do environmental factors influence this increase in transmission during Q4 and Q1? Is there more human-human transmission during this time, or is there more direct rodent contact? Please provide rationale for this surge.

Response: We have reviewed to raise the possible role of environmental factors in observed seasonality.
Additionally, please provide a description and a possible rationale for the uneven distribution of cases among professions. For example, are there more civil servants, students, and traders in general in the population, or is there actually a higher rate of exposure in these groups specifically?

Response: We have hypothesized possible reason for variation in proportions affected by occupation.

Reviewer #4: The authors have done well in retrospectively analyzing reported Lassa fever cases in Nigeria, their work will be useful in guiding policy makers towards making decisions on preparedness plans. I have found their language to be clear and unambiguous. There are however some concerns that they need to address as outlined below:

Line 73, who commenced this comprehensive documentation in SORMAS? States, Federal, hospitals?

Response: This has been addressed in the background section.

Line 107, why the use of different data sources? What is the role of SORMAS as stated earlier in line 73?

Response: The data sources mentioned are parts of one single surveillance data; they are complementary. Merging and harmonizing ensured we get the most complete data set. We mention these sometimes we have data on confirmed case for which part of the case data might be missing as with all secondary data. It is important we mention this to explain the slight differences in the denominators in some of the figures and/or tables.

Statement in lines 119-120 doesn’t align with what was earlier states in lines 103-7

Response: We have reviewed to provide more clarity on data source(s) and analyses performed. And merged the sub-sections: ‘Data Source’ and ‘Study Design’

Line 130, was there any reason why the case definitions on the IDSR were not used?

Response: The case definitions used for Lassa fever surveillance and response in Nigeria is based on the IDSR guidelines. This has been reflected clearly in the reviewed manuscript.

Line 154, is this a standardized definition or was it adapted from the preparedness guideline?

Response: This is for the purpose of this study to enable discussion of the findings and have been clearly stated in the revised manuscript.

In Table 2, is the N still valid for the occupation, state of residence and date of quarter in report on said table?

Response: Yes, it remains valid. In this table, the numbers represent the number of deaths while the percentages in the brackets represent the CFR (deaths/confirmed). We did not reflect the number of confirmed cases in this table so that it is not too busy and distract from the primary objective of the table – CFR. The whole numbers were provided in Table 1.

Line 199, the paragraph talks about monthly distribution, it will be good to reflect when the highest outbreak occurred in line 199 with respect to month and not year
Response: We did not reflect the month the highest month the outbreak peaked because it varied from one year to another. It is easier to reflect/discuss on the waves, when they begin and when they end, factors that influence the numbers and case fatality, as well as potential public health interventions. This has been the thrust of this paper, and earlier works on Lassa, COVID-19, among others.

In line 200, the use of epi week as against the earlier monthly distribution may be misleading, except a clear description of how epi weeks first relates to months is given

Response: The epi weeks used (4-10) were consistently from the end of January to the beginning of March in all the years under study (2018-2019). We have added a subsection “Epidemiological week’ under the ‘Methods’ section to define/clarify what an epidemiological week is.

In line 207 while talking of low numbers contributed by pupils/children, Is this low number in comparison to any subset? Better to compare age differently and describe findings from there and do same separately for occupation to avoid misinterpretation
Response: This is a valid observation. We have reviewed to limit comment on the occupational groups with high numbers and eliminated the comment on pupils/children sub-group considering that there are other sub-groups with low numbers. We added a separate interpretation on the age groups as advised.

Line 219, did you merge the age groups?
Response: This sentence has been deleted as it has been addressed by the response to the comments age distribution above.

Line 224, reconfirm the comment on slight increase in relation to figure 2
Response: This is a valid observation. We have corrected this typographical error to align. The sentence now aligned with our discussion points on the variation in case positivity rates under the ‘Discussion’ section.

Line 232 for CFRs, charts were in epiweek and results should follow same pattern, except if the authors would firstly define the epi weeks as they align with different quarter of the year
Response: This has been addressed in response to earlier comments on lines 199 and 200.

Lines 238-9, the number of affected states and fig 4a and 4c seem not to tally
Response: This is a valid observation. We have reviewed to reflect the correct tally for the states affected

Line 251, did it return to or it was lower than the geographical distribution of 2018?
Response: This is a valid observation. We have reviewed to limit the decline toward 2018 levels t0 the number of cases and rightly stated reduced geographic distribution compared to 2018

Lines 295-6, does the IDSR system not provide for probable cases and are they not reported in the SORMAS? You earlier gave a definition of probable cases, were there no probable cases reported within the review period?

Response 1: We did not find probable cases in our analyses

Response 2: We limited our analyses and conclusion to confirm cases to avoid confusion in whether we should add the probable cases to the suspected (denominator) or confirmed (numerator for case positivity; denominator for CFRs) cases in our analyses.

**General comments**:
The paper brought out further evidence of the recent changes to the epidemiology of Lassa in the country, particularly it increased all year-round incidence, and a new perspective on the differences in risk groups. Furthermore, being a review of country level secondary data, the paper stimulates further research interests based on some interesting hypothesis.

I believe the paper can be improved by having a paragraph in the background on some prevailing practices in Nigeria which have been linked to increased risk of spread of the disease epidemiology (open drying of grains, backyard farming, poor IPC practices, etc.).

Response: We have added a paragraph in the background on some prevailing practices in Nigeria linked to increased risk of Lassa fever spread.

A general review of the background paragraphs would be helpful, particularly in linking it to the study justification: it is understandable, but the flow could be improved in places.

Response: We have reviewed the background section to improve the flow and linkage to the study justification.

The data source section did not highlight how representative that data is, and if there are some assumptions related to the data sources and what mitigation measures were taken to minimize the effect on the conclusions driven from the results.

Response: We have reviewed the data source to highlight how robust and representative the data is and stated how we decreased the possibility of misclassification affecting validity of our conclusions.

The discussion section could be improved by highlighting possible explanation of some of the results (variations in geographic distribution, CFR, etc.). This will further strengthen some of the recommendations proffered.

Response: We have reviewed the discussion section to further hypothesise possible reasons for variations in geographic distribution and CFRs.

**Specific issues:**

Lines 49-52. The references seem outdated, with the stated epidemiology not in concurrence with what some recent data reveal.

Response: This has been reviewed to reflect more recent references and data.

Line 91-92. You have stated the role of the state and the federal government in health care and surveillance services but did not outline. That of the local government, which has the larger role in coordinating collation of surveillance data from all health facilities.

Response: The sentence has been reviewed to include the local government as enshrined in the National Technical Guidelines on Integrated Diseases Surveillance and Response.

Line 171-172. Review the sentence. There seems to be a grammatical issue there.

Response: This has been reviewed as appropriate.

Line 284. The paragraph is bulleted, which is likely a typographic error. Please review.

Response: It was a typo and has been reviewed.

Line 302-304. The sentence is vague and could be improved by paraphrasing some sections.

Response: This has been paraphrased as appropriate.

**Topic: Epidemiological Trends of Lassa Fever in Nigeria, 2018 -2021**

**Summary:** The author has described the trends of Lassa fever in Nigeria in the recent years to enhance the public understanding of its epidemiology and possible intervention strategies needed to mitigate the disease burden. The authors analyzed the national surveillance data on the disease obtained from the Nigeria Centre for Disease Control (NCDC), by describing the disease prevalence, sociodemographic, geographic distribution, and the clinical implications of the disease. As a result of this study, the authors recommend as a priority, the sustainability of improved surveillance, enhancement of laboratory diagnosis, and improvement of case management capacity during off-peak periods. Lassa fever is one of the deadly endemic diseases that are of human health burden in some African countries, as a result, studies like this are needed to provide more knowledge on the spread and control of the infection in the human population. With this being said, I recommend this work for publication in this reputable journal after some minor corrections.

**Background - Page 2**

1. Line 25: The first statement should be reconstructed. Are the authors intending to say that **Lassa fever is a viral hemorrhagic fever which is endemic in Nigeria**?

Response: This has been reconstructed as advised.

2. Line 25-Line 26: The second statement from Line25 should be rephrased. The authors need to be conscious of the sentences meaning. Here, the intention is to inform readers that improved surveillance and testing capacity has revealed an increase in number of Lassa fever cases. But the sentence here is indicating that it has resulted in an increased number of cases, which is not true.

Response: The sentence has been reviewed to provide more clarity as advised.

**Introduction – Page 3-4**

The author should revise the complete manuscript for typos, grammatical errors, and out-of-context sentences.

Response: The whole manuscript has been revised and proofread by the authors to address typos, grammatical errors and out-oof-context sentences as advised

For examples

1. Line 45: The first sentence is incomplete. Emphasize should be made on Lassa fever as an endemic disease, so include “tropical disease which is endemic in West Africa”

Response: The paragraph has been reviewed to provide more clarity.

2. Line 47: Include the full meaning of RNA before the abbreviation.

Response: The full meaning of RNA has been inserted before the abbreviation as advised.

3. Line 47 – Line 49: This is a long sentence that will need some punctuation marks.

Response: The sentence has been broken into two and provided better punctuation.

4. Line 60: Include the abbreviation in a bracket like (ILFR&C), followed by a comma

Response: This has been done in the reviewed manuscript.

5. Line 65 – Line 67: This is a long sentence that will need some punctuation marks.

Response: The sentence has been broken into two and provided better punctuation.

6. Line 81 – 83: The authors should use this section to emphasize on the novelty of this study. There have been literatures on Lassa fever trends, how is this study different and what is the advantage of this study to present knowledge on the disease burden in Nigeria.

Response: The section has been reviewed to emphasise the novelty of the study and why it provided better evidence to enable improvement in surveillance and response for Lassa fever.

**Page 5**

1. Line 98: I will assume the Study design was meant to be a subsection like other are. I will suggest that the Study design and data source should be merged since the writeup in the study design is so limited.

Response: The two subsections have been merged as advised.

2. Include the full meaning of IDSR before the abbreviation, also in Page 6 Line 126, the LF should be defined before abbreviation.

Response: The two abbreviations have been addressed as advised

**Results – Page 8**

1. Line 180 – 181: The authors reported that CFR had significant improvement over the years in high-burden states except for 2021. WHY? Authors should discuss possible cause of this result. This can be discussed from the standpoint of the reported cases, is there any difference in the trends? The epidemiological implications should also be discussed.

Response: This has been discussed under the discussion section hypothesizing two possible reasons:

i. Healthcare workers more proficient in high-burden staes
ii. The deployment of more human and financial resources by the state and national authorities whenever there are high cases reported including activation of EOC/IMS structures

An additional reason, the fact that there is an Institute of Lassa Fever Research and Control (ILFR&C), Irrua Specialist Teaching Hospital (ISTH) within or in close proximity to the high-burden States has been added to the discussion section.

**Discussion**

1. Line 256: COVID-19 not COVID 19.

Response: This typo has been corrected

2. The authors have recommended some further studies requirements and implementations that can further reduce the burden of this disease in the human population. However, it is also important to investigate the impact of control interventions and the financial resources available for a response particularly during outbreaks. As a result, the authors should include and refer readers to the following studies on Lassa fever modeling in Nigeria.

- Mathematical analysis of a Lassa fever model in Nigeria: optimal control and cost-efficacy. *Int. J. Dynam. Control* (2022).
- Optimal control and cost-effectiveness analysis for the dynamic modeling of Lassa fever, J. Math. Comput. Sci., 12 (2022), Article ID 136
- Modeling, analyzing, and simulating the dynamics of Lassa fever in Nigeria. *J Egypt Math Soc* **30,** 1 (2022).
- Modeling the dynamics of Lassa fever in Nigeria *J Egypt Math Soc* **29**, 16 (2021).

Response: This valid observation has been addressed

**General comments**

The author needs to read and revise the complete manuscript as this cannot be accepted in this version. I will recommend this for publication after making the above corrections and suggestions.

Response: All the comments were relevant; we have tried as much as possible to address them. We thank the reviewers for their thorough review.
